# Vulnerability of South African women workers in the COVID-19 pandemic

**DOI:** 10.3389/fpubh.2022.964073

**Published:** 2022-09-09

**Authors:** Naidoo Saloshni, Naidoo Rajen Nithiseelan

**Affiliations:** ^1^Discipline of Public Health Medicine, School of Nursing and Public Health, University of KwaZulu-Natal, Durban, South Africa; ^2^Discipline of Occupational and Environmental Health, School of Nursing and Public Health, University of KwaZulu-Natal, Durban, South Africa

**Keywords:** COVID, working women, vulnerability, socio-economic, South Africa

## Abstract

On March 5th, 2020, the first SARS-CoV-2 (COVID-19) case was diagnosed in South Africa. Shortly after, President Cyril Ramaphosa, declared a National State of Disaster placing the country under “lockdown”. Two years later the National State of Disaster was terminated on 15 March 2022 with more than 3.9 million cases of COVID-19 and more than 100,000 fatalities recorded. In the context of this pandemic the vulnerability of working women in South Africa increased considerably. In South Africa most women workers find themselves in vulnerable employment as domestic help in private households, traders in the informal economy, and small-scale agriculture with no employment contracts or health insurance cover. During the pandemic, women workers had to further deal with the socioeconomic vulnerability of their employment, dual domestic and working responsibilities and those infected with COVID-19, with the clinical sequelae of the disease. The government implemented several policies to assist workers and reduce the risk faced by vulnerable workers, including women. Despite these initiatives, long-term policies aimed at socioeconomic protection and employment creation that focus on women workers are required to address the negative impact of the COVD-19 pandemic as experienced by women workers in South Africa.

## Introduction

The SARS-CoV-2 pandemic is in its third year with more than 600 million infections and in excess of 6 million deaths globally (1) and far-reaching socio-economic consequences for people in all countries. On March 5th, 2020, the first SARS-CoV-2 (COVID-19) case was diagnosed in South Africa. Shortly after, the President declared a National State of Disaster placing the country under “lockdown”, limiting movement to purchase of essential items and access of emergency care (2). Two years later, with declining daily death rates, the National State of Disaster was terminated on 15 March 2022. More than 3.9 million cases of COVID-19 and more than 100,000 fatalities have been recorded in a population of ~60 million (3). The country has experienced five waves of COVID-19 infections with the Delta and Omicron variants of COVID-19 driving the severe second and fourth waves in the country (4, 5). Owing to the recurrent peaks in the burden of COVID-19 infections, the country has returned to different Alert Levels and lockdown at various points in the pandemic. During Alert Level 5, drastic measures were taken, with closure of all sectors except for those providing essential services such as health, food production and sales. Confined to their homes, people were only permitted to go out to purchase food and access essential services. Between Alert Levels 4 and 2 there was an easing of the restrictions but several remained in place. Alert Level 1 allowed for ease of movement and opening of all sectors in the country, with limits on gatherings and continuation of the public health principles of physical distancing, hand sanitizing and the legislated use of facemasks in public spaces (6).

All of these restrictions have had implications for the population at large and workers in particular, whose vulnerability has increased in this pandemic. In this paper, we explore the impact the COVID-19 pandemic has had on women workers in South Africa and provide a perspective on addressing this impact in women workers in the country.

## Employment and economic vulnerability of women workers

South Africa, like most low and middle income countries globally, has formal and informal employment sectors, with 63.8% of employed South African's working in the formal sector in 2021. Unemployment levels during the pandemic increased from 32.6 to 34.4% in 2021. In the 2nd quarter of 2021, ~32.4% of women were employed in the country. Of those women who were employed ~68% were in formal sector employment while 14.6, 13.5, and 3.9% worked in the informal sector, private households and agriculture. Most women working in the formal sector in 2021 worked in jobs in community and social services (including healthcare) (32.7%), trade (21.5%) and finance (13.7%), respectively (7).

The informal sector in South Africa comprises employees working in establishments employing less than five employees or employers, own account holders or persons helping in their own household business who are not registered for income tax or value-added tax. This sector consists of a vast array of enterprises including but not confined to street vendors and hawkers, “spaza” shops (township shops), hairdressers and barbers, food outlets, garment manufacture, mechanics and panel beaters (8). Fractionally more women (50.8%) work as “own account holders” in the informal sector as compared to men (49.2%) in South Africa and more women aged 35 years and above tend to find themselves working in the informal economy (9).

Women workers in South Africa are far more economically vulnerable when compared to their male counterparts. Most women in South Africa, whether employed in the formal or informal sector find themselves in low skilled and poorly paying jobs. Often these jobs are contractual in nature with no basic benefits such as pensions and medical insurance (10). As a result, when the economy faces a crisis as has been case with the COVID-19 pandemic, they are more likely to lose their jobs and experience financial insecurity. Not surprisingly, unemployment amongst women in South Africa saw a steady increase from 31.3% in the 2nd quarter of 2019 to 36.8% in the 2nd quarter of 2021 (7, 11). The South African National Income Dynamics Study Coronavirus Rapid Mobile Survey conducted between May and June 2020 found that women in the informal economy experienced a decrease in their working hours by as much as 49% and those women in informal self-employment reported a decrease in income by as much as 70% (12). These decreases in income increased the already vulnerable position of working women in South African society.

## Burden of COVID-19 infection in women workers

Globally the number of COVID-19 cases in women (52%) exceeds that of men (48%) despite mortality being worse in men (1). This pattern is replicated in South Africa with current infections in women accounting for more than 55% of the total burden of COVID-19 infections in South Africa (3). Owing to the COVID pandemic a national occupational health surveillance system monitored by the South African National Institute of Occupational Health has been implemented requiring employers to register on the system and report workers diagnosed with COVID-19. The May 2020 COVID-19 transmission by Occupation report from this surveillance, found that women [median age of 40 years, interquartile range (IQR), 19–86 years] accounted for 56% of COVID-19 infections in the workplace for these cases with available occupational information (13). The industries mainly reporting information were from services and sales, health care and management. Undoubtedly, women in the healthcare sector in South Africa have been at the coalface of the pandemic. As of November 2021 of the more than thirty-nine thousand COVID-19 hospital admissions, 2.4% were health care workers. Female health care workers accounted for 67% of all admissions amongst health care workers (14). Increased infections amongst women workers and slow recovery amongst those with severe morbidity delayed return to work and increased the vulnerability amongst these working women.

## Psychosocial impact of COVID-19 on women workers

There is no doubt that working women globally experienced a significant psychosocial burden during the pandemic. The sources of stress felt by women workers were multiple. These included loss of employment and decreases in income, the risk of COVID-19 infection, working longer hours and gender based violence (15).

In South Africa the risk of contracting COVID-19 or transmitting the disease posed a stress to several workers. Those who worked in jobs interacting with the public such as in health and services worried about taking infection home to family (16). An online survey of South African health care workers experiences of COVID-19 conducted between April and May 2020 in the country found that amongst female health care workers more than 20% (95% CI 19.7–23.8) were severely distressed (17).

Working longer hours increased stress in women workers. Those women who continued to go to work had to work longer hours when colleagues contracted COVID-19. Health care workers in particular started to experience burnout owing to stressful conditions, the longer hours and reduced time-off they faced during the pandemic (18). The experiences of healthcare workers during the pandemic in South Africa are similar to that of healthcare workers the globally (19–21).

South Africa is a patriarchal society in which, the vulnerability of women workers is further increased by virtue of the additional responsibilities they face on the home front in the form of domestic work, caring for children and the elderly. During lockdown most women found that in addition to working from home, they had the added burden of caring for family members who were ill, monitoring children who were having online schooling and general household chores. This resulted in blurring of the lines between work and home responsibilities. There were also those women who had to go to their workplaces and then return to the added responsibilities, worsening the psychosocial stress they experienced. A survey amongst 185 informal sector workers in Durban, South Africa found that women reported far greater increases, of between 37 and 59%, in cooking, cleaning and child care responsibilities when compared to their male counter-parts (21–27%) (22).

Women who were the sole financial support in their homes experienced increased stress owing to loss of earnings. In 2019, 41.8% of South African households were female-headed (23). The UNDP report on the socio-economic impact of COVID-19 on South Africa indicated that female-headed households were more likely to fall into poverty and persist in this state during the pandemic as compared to male- headed households (24).

Reported cases of gender-based violence increased considerably in South Africa during lockdown. Women either working from home or being unemployed found themselves with increased exposure to their violent partners (25). Based on the 2022 report from the South African Police there was a 10% increase in sexual offenses when comparing the financial period April to June 2017 /2018 (*n* = 11,526) to the same period in 2021/2022 (*n* =12,702) (26). The increase in gender-based violence added to psychosocial stress experienced by women during the pandemic in South Africa.

## Interventions

The government implemented several policies to assist workers and reduce the risk faced by vulnerable workers, including women. Regulations and workplace guides were implemented to reduce the risk of COVID-19 transmission in the workplace and identify vulnerable workers, for intervention (26–31).

The workplace guides while initially focused on the healthcare setting expanded to include all sectors such as manufacturing, mining and transport. These guides focused on identifying COVID-19 risk in the workplace, reducing exposure through improved ventilation and infection prevention and control practices and managing the COVID-19 positive worker (29–31). COVID-19 infections acquired in the workplace were declared compensable, requiring employers to report cases to the Compensation Commissioner (27). Unfortunately all of these interventions applied to the formal sector and could realistically only be implemented in the formal work sector. Practices of infection prevention control for COVID-19 were difficult to implement in the informal sector where basic access to water and sanitation remain a major limitation. However, there were non-governmental organizations such as Asiye eTafulani in the eThekwini municipality who in collaboration with WIEGO and public health experts produced health and safety guidelines and handwashing stations to help street traders. Importantly though in the main traders were responsible for purchasing their own personal protective equipment which increased the financial burden they were already experiencing (22).

The South African government announced several financial interventions aimed at providing social protection for South Africans in the midst of the pandemic. The COVID-19 Temporary Employee/ Employer Relief Scheme (”TERS”) was announced in March 2020. This scheme allowed for the provision of supplementation income for employees who had a reduction in their salaries due to reduced working hours (32). Unfortunately, this scheme only covered employers and employees in the formal sector. Women working in the informal sector did not qualify for the benefits of this scheme.

The child support grant was increased for a month and then converted to a Caregiver allowance paid to the primary caregiver of children who received the child support grant from June to October 2020. Often in South Africa, children are left to the care of grandmothers in rural communities while women travel to urban areas for work and so this incentive while benefitting the household in which the child resided, would not benefit an unemployed woman unless she was the primary caregiver for her child.

A COVID-19 Social Relief Distress Grant was paid for a period of 9 months to individuals who were unemployed and ineligible for other grants or unemployment benefits (12). This grant continues in 2022. For a period prior to the Social Relief Distress Grant, food aid from the South African Social Security Administration was distributed through municipal structures (12). Women who received child support grants were excluded from this incentive. As a result, several women in the informal sector who received child care grants were excluded (12). Further the applicants details were cross-checked through seven databases including, Home Affairs, the South African Revenue Services and government salary prior to awarding of the grant. This delayed the process for recipients considerably (12). In addition women who were migrants or asylum seekers were excluded as they were not considered for financial support through the COVID-19 Social Relief Grant (33).

**Figure 1:**
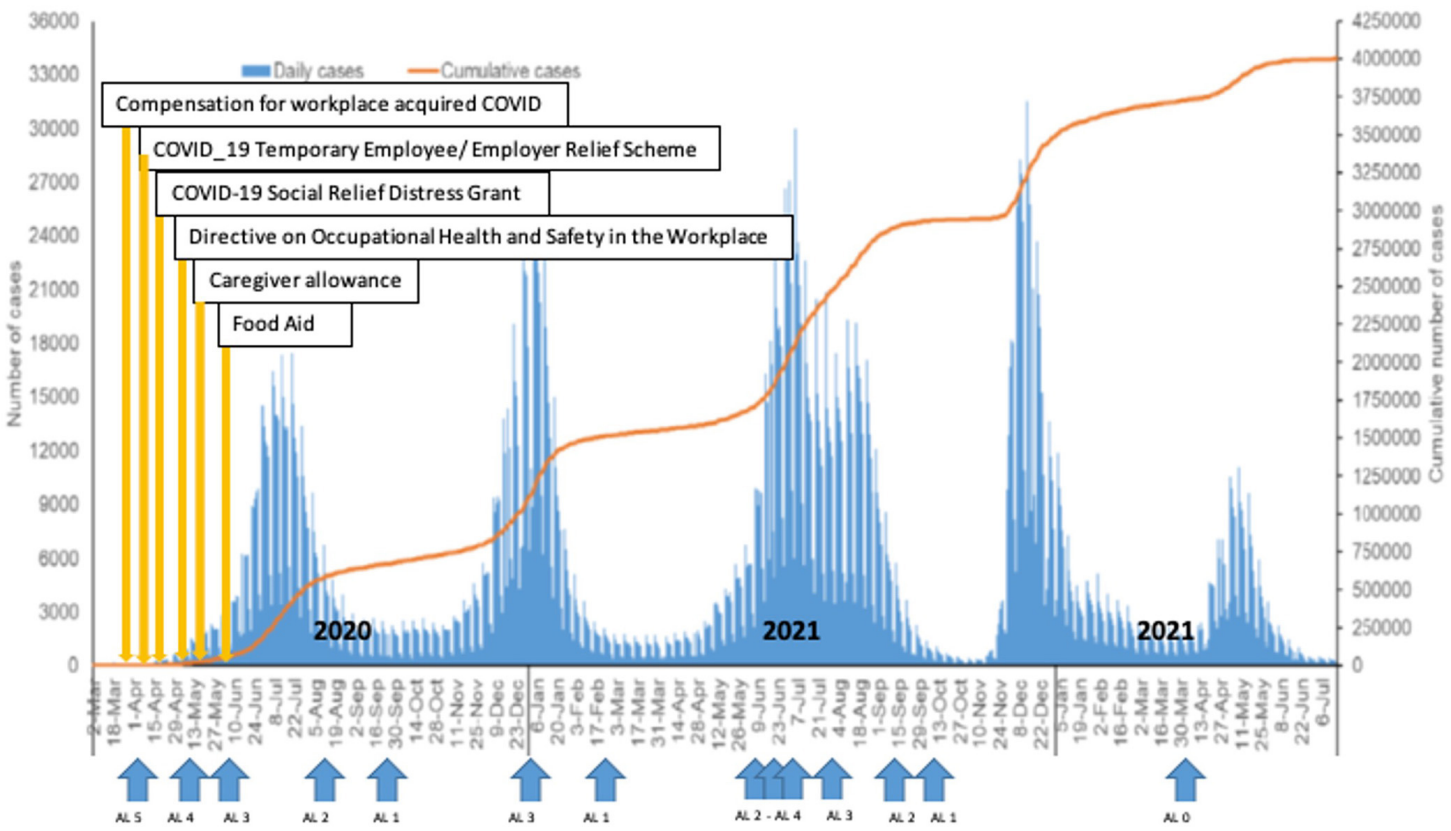
South African COVID-19 Alert levels (35) and dates of specific interventions. AL = Alert Level.

While some informal sector workers were able to access the social relief initiatives there were challenges for others. An absence of identification documents, no access to digital services and a lack of bank accounts were amongst the challenges experienced by the workers. All applications for social relief had to be made digitally and in the absence of data or WiFi connectivity, this could not be done. Further, ~20% of South Africans do not have bank accounts (34) and the grants were paid into bank accounts to avoid large queues at pay-points, which would have been a contravention of lockdown rules and a challenge for infection prevention and control. Hence those who did not have bank accounts were disadvantaged (Figure 1).

## Discussion

While there were both workplace and social interventions implemented aimed at assisting workers, the complicated processes required to achieve the benefits of these interventions proved frustrating for workers and often excluded those most in need such as women workers increasing their vulnerability. The existing vulnerability of working women in South Africa worsened during the COVID-19 pandemic through job losses, increased risk at work and domestic pressures. The current measures aimed at assisting working women are only interim measures and long-term policies aimed at socioeconomic protection and employment creation that focus on women workers are required to address the negative impact of the COVD-19 pandemic as experienced by women workers in South Africa.

The national occupational health surveillance system that was implemented needs to be supported and extended to other aspects of worker health and to ensure that women workers are included. Further working with local authorities and non-governmental organization surveillance has to be extended to include the informal sector where most women workers find themselves in South Africa. This will require investment in systems and human resources.

The International Labor Organization in 2015 issued Recommendation 204 concerning the “Transition from the Informal to the Formal Economy”. This recommendation provides the guiding principles for countries to embrace when designing strategies to formalize the informal economy. This requires necessary policy frameworks be implemented (36). In South Africa amongst the policies extending unemployment insurance to women in the informal sector and those employed in temporary contracts will aid in filling the gap that currently exists with respect to this system. Women in the informal sector are organized in informal groups (such as domestic workers) and linked to non-governmental organizations, which can be used to link them to the unemployment insurance fund. Identifying easier ways for women to access financial support, be it grants or funding for small businesses, is crucial to ensuring the money and support reaches those most in need of it. Due consideration should be given to increasing the child support grants and converting the COVID-19 relief grant into a permanent basic income support grant available to all South African earning below. Further, the current amount of R350 should also be increased to a livable amount.

Greater attention is needed to encourage job security for women workers in South Africa, with concerted efforts to move away from contractual jobs to greater job permanency. Supportive work environments which encourage skills development and up-skilling giving women the opportunity to compete with their male counterparts for permanent positions are required. These environments need to take cognizance of the dual roles women play in the workplace. Further, the impact that COVID has had on women's health both physically and mentally has to be dealt with in the workplace through worker health programs. Programs to support women's health in the informal sector can be implemented with support from NGOs and ward based outreach teams at a primary health care level.

**Figure 2:**
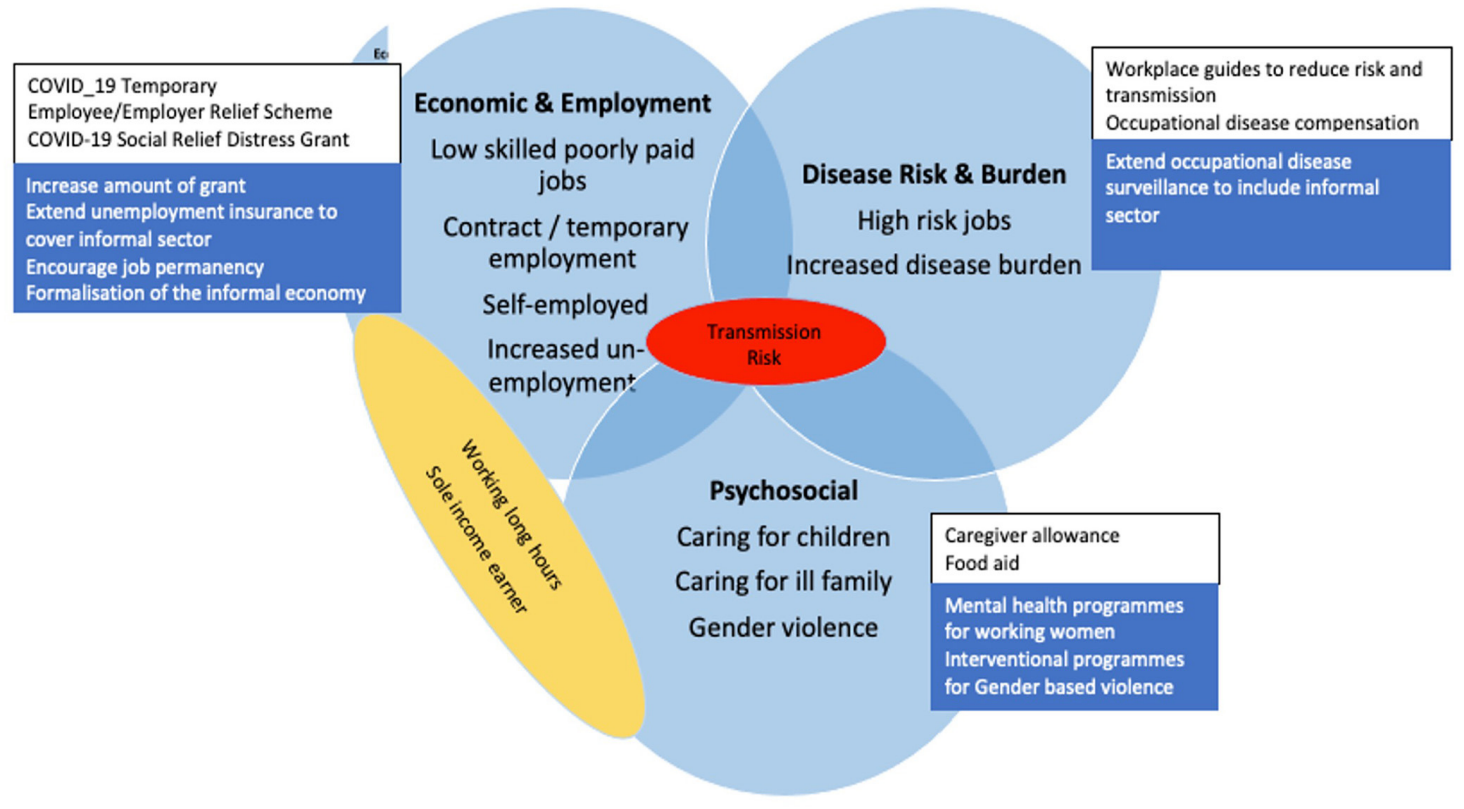
Vulnerabilities, Government interventions and proposed recommendations for addressing the COVID-19 impact on South African women workers. Blue circles: vulnerability factor, red oval: transmission risk, yellow oval: overlay of economic and psychosocial vulnerabilities, white box: government intervention, blue box: recommendations.

In South Africa, gender-based violence had been recognized as a human right violation which is a first step in addressing this problem in society. However, uplifting women' socioeconomic status empowers them to make their own choices. This is a very important step in addressing gender-based violence and due attention should be given to it (Figure 2).

In summary a comprehensive program addressing the short-term and long-term impacts of COVID-19 on women workers in needed in the current South African context. Such a program will have to be flexible and resilient in the changing South African work context.

## Data availability statement

The original contributions presented in the study are included in the article/supplementary material, further inquiries can be directed to the corresponding author/s.

## Author contributions

NS conceptualized and wrote the paper including revisions. NN reviewed and commented on all drafts of the paper. All authors contributed to the article and approved the submitted version.

## Conflict of interest

The authors declare that the research was conducted in the absence of any commercial or financial relationships that could be construed as a potential conflict of interest.

## Publisher's note

All claims expressed in this article are solely those of the authors and do not necessarily represent those of their affiliated organizations, or those of the publisher, the editors and the reviewers. Any product that may be evaluated in this article, or claim that may be made by its manufacturer, is not guaranteed or endorsed by the publisher.
